# Tissue Distribution of Porcine FTO and Its Effect on Porcine Intramuscular Preadipocytes Proliferation and Differentiation

**DOI:** 10.1371/journal.pone.0151056

**Published:** 2016-03-10

**Authors:** Xiaoling Chen, Bo Zhou, Yanliu Luo, Zhiqing Huang, Gang Jia, Guangmang Liu, Hua Zhao

**Affiliations:** Key Laboratory for Animal Disease-Resistance Nutrition of China Ministry of Education, Institute of Animal Nutrition, Sichuan Agricultural University, Chengdu, Sichuan, 611130, P. R. China; University of Bologna, ITALY

## Abstract

The fat mass and obesity associated (*FTO*) gene plays an important role in adipogenesis. However, its function during porcine intramuscular preadipocyte proliferation and differentiation remains poorly understood. In this study, we prepared the antiserum against porcine FTO (pFTO), which was used to determine its subcellular localization and tissue distribution. Our data indicated that pFTO was localized predominantly in the nucleus. Real-time quantitative PCR and western blot analysis showed that pFTO was highly expressed in the lung and subcutaneous adipose tissue. Overexpression of pFTO in porcine intramuscular preadipocytes significantly promoted cell proliferation and lipid deposition. Furthermore, overexpression of pFTO in differentiating porcine intramuscular preadipocytes also significantly increased the mRNA levels of adipocyte differentiation transcription factors peroxisome proliferators-activated receptor γ (PPARγ), CCAAT/enhancer binding protein α (C/EBPα), lipoprotein lipase (LPL) and fatty acid synthase (FAS). Our findings provide the first functional evidence to reveal a role of pFTO in porcine intramuscular preadipocyte proliferation and differentiation.

## Introduction

Pork quality is becoming a major concern for the swine industry due to the development of export markets and increased consumer demands [1]. Intramuscular fat (IMF) is one of the important factors influencing meat quality, which shows a positive correlation with the meat quality traits such as the flavor, tenderness, juiciness and water holding capacity [2, 3]. IMF content could be modulated by some key genes [4]. Thus, it is necessary to identify candidate genes related to IMF content in the pig.

In 2007, the fat mass and obesity associated (*FTO*) gene was discovered and found to be significantly associated with both children and adults obesity [5]. A genome-wide study showed that the *FTO* gene was located on procine chromosome 6 [6], which probably hosts genes related to meat quality traits including IMF content [7, 8]. In addition, the porcine *FTO* (*pFTO*) gene has been widely reported to be associated with some fat-related traits [9–17], including IMF deposition, IMF content or total lipid percentage in muscle in pigs.

In this study, we prepared the polyclonal antibody against pFTO and detected its tissue distribution and localization. We also investigated the role of pFTO in porcine intramuscular predipocytes proliferation and differentiation.

## Materials and Methods

### Ethics statement

This study was carried out in strict accordance with the recommendations in the Guide for the Care and Use of Laboratory Animals of Sichuan Agricultural University. The protocol was approved by the Animal Care Advisory Committee of Sichuan Agricultural University under permit No. YYS130125.

### Antibody production and identification

Polyclonal antibody against pFTO was prepared as described previously [18]. Initially, 300 μg of the purified recombinant pFTO protein [19] was multi-point injected (subcutaneous) into the Kunming mouse after being emulsified with complete Freund’s complete adjuvant. After 2 weeks, the booster injection was administered with the same amount of antigen emulsified incomplete Freund’s adjuvant. The booster injections were repeated three times in a 10-day interval, and the antiserum was collected at 7 days after the final booster injection. ELISA analysis was performed according to methods described by [18].

### Tissue sample collection

Three 10-week-old female Duroc × Yorkshire × Landrace (DLY) pigs (31–31.6 kg body weight) were slaughtered in a humane manner according to protocols approved by the Animal Care Advisory Committee of Sichuan Agricultural University (Permit Number: YYS130125). The tissue samples of liver, heart, skeletal muscle, lung, kidney, spleen, and subcutaneous adipose were removed and immediately snap-frozen in liquid nitrogen before being stored at -80°C until use.

### Plasmid construction

The DNA fragment containing the entire open reading frame of *pFTO* was amplified using primers FTO-PCDNA3.1(+)-Hin-F (5’- CCCAAGCTTATGAAGCGAACCCCAACCGCCG-3’) and FTO-PCDNA3.1(+)-Xho-R (5’-CCGCTCGAGCTAGGGTTTGGCTTCCAGAAGC-3’) and the plasmid pET30a(+)-pFTO [19] as a template. The FTO-PCDNA3.1(+)-Hin-F and FTO-PCDNA3.1(+)-Xho-R primers introduced *Hind*III and *Xho*I restriction sites (underlined), respectively. The purified PCR product was digested with *Hind*III and *Xho*I and ligated into pcDNA3.1(+) digested with the same restriction enzymes. The recombinant construct was transformed into *E*. *coli* DH5a strain and kanamycin resistant colonies were selected. Proper construction was identified by colony PCR and DNA sequencing and was designated as pcDNA3.1(+)-pFTO.

### Cell isolation and culture

Porcine intramuscular preadipocytes were isolated from longissimus lumborum muscle of DLY piglets less than 3 days old. The pig obtained from Sichuan Zhengyuan swine industry Co., Ltd. (Chengdu, Sichuan, China) was slaughtered according to protocols approved by the Animal Care Advisory Committee of Sichuan Agricultural University. Briefly, approximately 1 g of the muscle was removed aseptically and washed in phosphate buffered saline (PBS) under sterile conditions. The muscle tissue was cut into pieces approximately 1 mm^3^ in size using ophthalmic scissors, transferred to 50 mL centrifuge tubes, and digested with 0.2% collagenase II (Sigma) for 2 h at 37°C in a water bath. The digested solution was filtered through 200 and 400-mesh cell sieves. The filtered solution was collected and centrifuged at 1,000 rpm for 10 min. The pellet was resuspended in DMEM/F12 medium supplemented with 15% fetal bovine serum (FBS) (Gibco), 100 U/mL penicillin, and 100 μg/mL streptomycin. Cells were maintained at 37°C under 5% CO_2_ in a humidified incubator. The medium containing unattached cells was discarded after 6 h and fresh culture medium was added. The culture medium was renewed every 2 days. The cells (passages 1–3) were identified by immunofluorescence with anti-DLK1/Pref-1 antibody (Santa Cruz Biotechnology, Santa Cruz, CA, USA).

Cell differentiation was initiated as before [20]. Briefly, two days after contact inhibition, the cells were induced to differentiation with the hormone cocktail [0.5 mM 3-isobutyl-1-methylxanthine (IBMX), 1 μM dexamethasone (DEX), 10 μg/mL insulin] for 2 days. The medium was then shifted to medium containing 10% FBS and 10 μg/mL insulin for 2 days, followed by replaced with DMEM/F12 supplemented with 10% FBS for the remaining culture period. The medium was replaced every 2 days.

### pFTO overexpression

Porcine intramuscular preadipocytes were seeded in 24-well plates and transfected with 0.5 μg of the plasmid pcDNA3.1(+)-pFTO or the empty vector pcDNA3.1(+) using Lipofectamine 2000 (Invitrogen) according to the manufacturer’s instruction.

### RNA isolation and reverse transcription

Total RNA was isolated from the adherent cultured porcine intramuscular preadipocytes and collected tissue samples using RNAiso Plus reagent (TaKaRa, Dalian, China). The concentrations of RNA were determined using a Beckman DU-800 spectrophotometer (Beckman Coulter, Fullerton, CA, USA). One microgram of total RNA was transcribed into single-stranded cDNA using PrimeScript^®^ RT reagent Kit with gDNA Eraser (TaKaRa).

### Real-time quantitative PCR

Real-time quantitative PCR was performed using an ABI 7900HT Real-time PCR system (384-cell standard block). The gene specific primers (Sangon Biotech, Shanghai, China) used are listed in Table 1. The PCR cycling conditions used were: 45 cycles at 95°C for 15 s and 60°C for 30 s. Data analysis was performed using the comparative Ct method [21] with *β-actin* as an endogenous control.

**Table 1:** List of genes, primer sequences, GenBank accession numbers, and product sizes in this study.

Gene name	Primer	Sequence	GenBank accession no.	Product size (bp)
*FTO*	Forward	5'-ACCTGAAAGAGGAGCCCTAC-3'	**KM232950**	170
	Reverse	5'-CCACACATCGGGATCTCTG-3'		
*PPARγ*	Forward	5'-AATTAGATGACAGCGACCTGGCGA-3'	**NM_214379**	102
	Reverse	5'-TGTCTTGAATGTCCTCGATGGGCT-3'		
*C/EBPα*	Forward	5'-CGTGGAGACTCAACAGAAGG-3'	**AF103944**	95
	Reverse	5'-GCAGCGTGTCCAGTTCGCGG-3'		
*LPL*	Forward	5'-ACCGTTGCAACAACTTGGGCTATG-3'	**NM_214286**	98
	Reverse	5'-ACTTTGTAGGGCATCTGAGCACGA-3'		
*β-actin*	Forward	5'-CCACGAAACTACCTTCAACTCC-3'	**DQ845171**	132
	Reverse	5'-GTGATCTCCTTCTGCATCCTGT-3'		

### Western blot analysis

Western blot analysis was performed as described by [22]. Briefly, proteins were transferred to a nitrocellulose membrane (Beyotime) after electrophoresis, and then the membrane were blocked with 3% non-fat milk in TBST. The membrane was incubated with the prepared mouse anti-pFTO polyclonal antibody followed by horseradish peroxidase (HRP)-conjugated goat anti-mouse IgG. The signals were visualized by using SuperSignal West Pico Chemiluminescent Substrate (Pierce) according to the manufacturer’s instructions.

### Immunofluorescence

Porcine intramuscular preadipocytes were cultured in 24-well plates. After washing twice with PBS buffer, cells were fixed with 4% paraformaldehyde in PBS for 15 min. Cells were then washed three times with PBS and permeabilized with 0.5% Triton X-100 in PBS for 20 min. After washing three times with PBS, cells were incubated with immunol staining block buffer (Beyotime) for 30 min and incubated at 4°C overnight with the anti-DLK1/Pref-1 antibody (1:100) or the prepared anti-pFTO polyclonal antibody (1:100). Cells were washed three times with PBS and subsequently incubated with the FITC-conjugated secondary antibody (1:200, Santa Cruz) in the dark for 1 h at 37°C. To detect the cell nucleus, specimens were further incubated with 4’,6’-diamidino-2-phenylindole (DAPI) (Beyotime) for 10 min. Background staining was removed by washing three times with PBS. Images were collected on a Nikon Eclipse TS100 inverted fluorescence microscope.

### EdU proliferation assay

EdU (5-ethynyl-2'-deoxyuridine), a nucleoside analog of thymidine, is incorporated into DNA during active DNA synthesis only by proliferating cells. Proliferating porcine intramuscular preadipocytes were determined by using the Click-iT EdU Alexa Fluor 594 imaging kit (Invitrogen) according to the manufacturer’s instruction. Briefly, porcine intramuscular preadipocytes were incubated with 10 μM EdU for 6 h before fixation, permeabilization, and EdU staining. Cell nuclei were stained with Hoechst 33342 (Invitrogen) at a concentration of 5 μg/mL for 30 min.

### Oil red O staining

On day 8 of differentiation, the lipid droplets present in porcine intramuscular preadipocytes were stained with the Oil red O staining kit (GENMED Scientifics INC. USA) as before [20]. Briefly, cells were washed with PBS and fixed with 4% formaldehyde in PBS for 1 h. Then the cells were washed with 60% propan-1-ol and stained with Oil red O solution for 20 min, followed by rinsed with distilled water before being destained in 100% propan-1-ol for 15 min.

### Triglyceride content Assay

Triglyceride (TG) content was analyzed using triacylglycerol detection kit (Nanjing Jiancheng Bioengineering Institute, Nanjing, China) according to the manufacturer’s protocol. Briefly, the differentiated porcine intramuscular preadipocytes were washed with PBS and subjected to sonication for rupture. BCA protein assay kit (Pierce) was used to measure the protein concentration of disrupted cells. The TG content was expressed in mmol/g protein.

### Statistical analysis

All data were expressed as mean ± SE (standard error). Data analyses were performed using SPSS11.5 software. Group differences were analyzed by One-way ANOVA followed by Tukey test. *P* < 0.05 was considered statistically significant.

## Results

### Titre and specificity of polyclonal antibody

**Fig 1:**
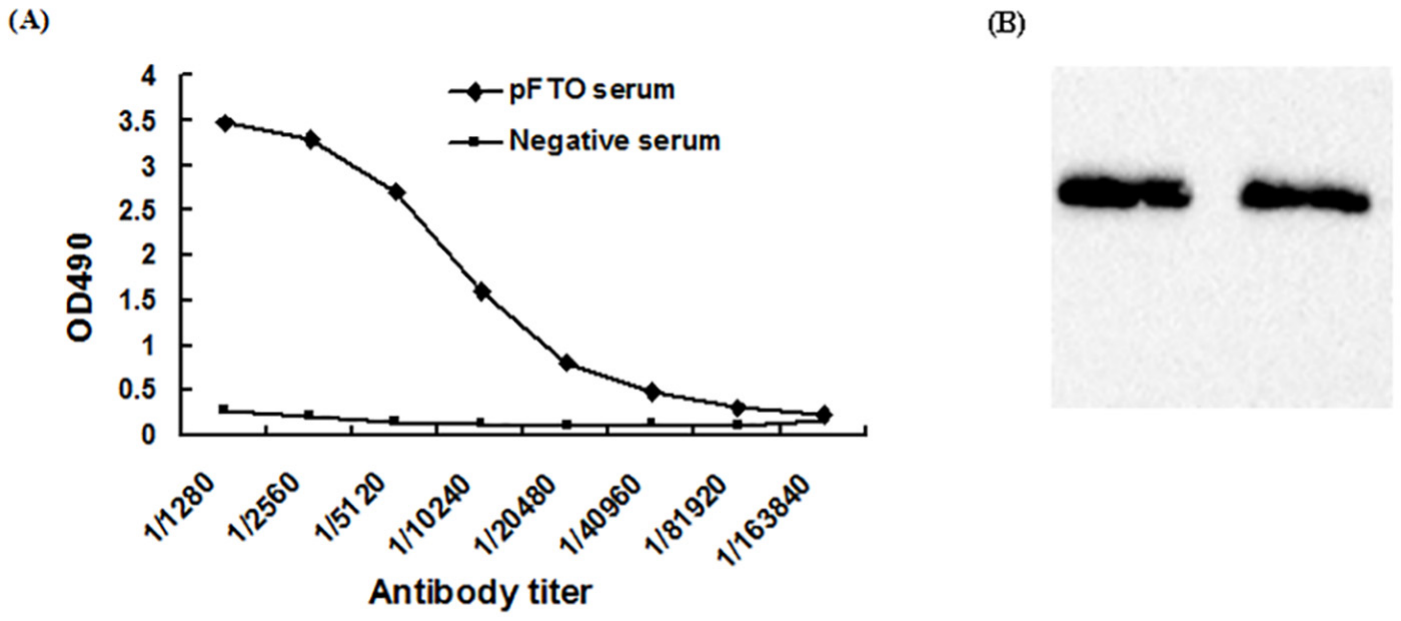
Identification of the polyclonal antibody against pFTO. (A) ELISA of the antiserum against pFTO; (B) Western blot analysis of anti-FTO antibody. Lanes 1 and 2: the purified pFTO protein detected by polyclonal antibody against pFTO.

FTO antiserum was diluted to different concentrations (1:1280–1:163840) and was detected by ELISA. The titre of the antiserum in ELISA test was 1:128,000 (Fig 1A), which was 2.1-fold greater than pre-immunization serum [23]. Western blot analysis revealed the antiserum obtained was specific to its immunogen, recombinant pFTO (Fig 1B).

### Tissue distribution of pFTO

**Fig 2:**
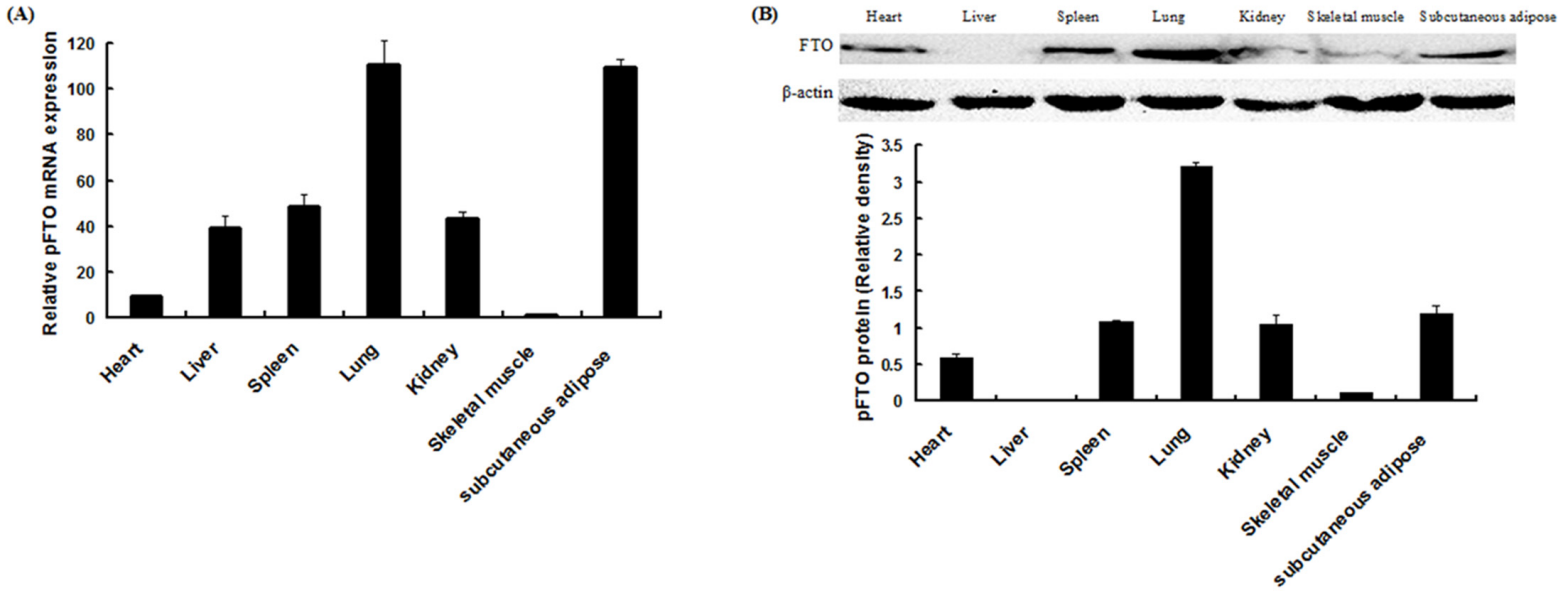
Tissue distributions of pFTO. (A) Relative expression levels of *pFTO* mRNA in different tissues. (B) Western blot analysis of pFTO protein levels in different tissues. The amount of *pFTO* was normalized to the amount of *β-actin*. Bars presented the means ± SE (n = 3).

Expression of pFTO was assessed by real-time quantitative PCR and western blot analysis in various porcine tissues. As shown in Fig 2A, pFTO mRNA was most abundant in the lung and subcutaneous adipose, followed by the spleen, kidney, liver, heart and skeletal muscle. As shown in Fig 2B, pFTO protein was most abundant in the lung, followed by the subcutaneous adipose, spleen, heart and kidney, and to a lesser extent in the skeletal muscle. No pFTO protein was detected in the liver (Fig 2B).

### Identification of porcine intramuscular preadipocytes

**Fig 3:**
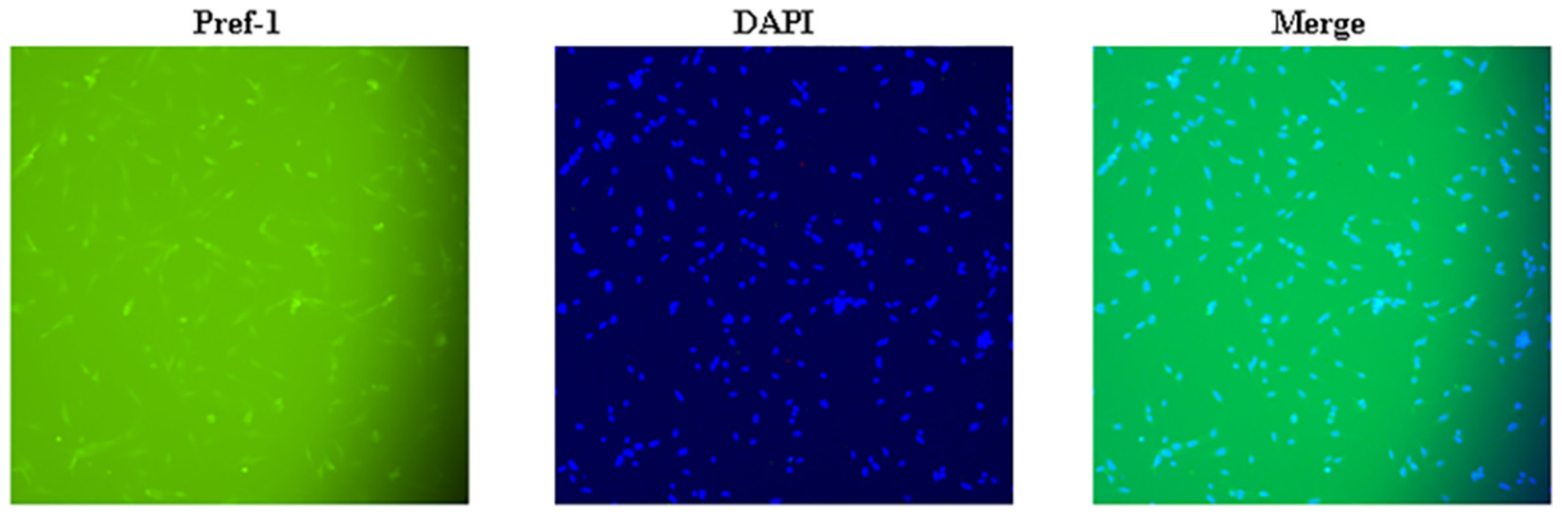
Identification of porcine intramuscular preadipocytes. Porcine intramuscular preadipocytes were characterized by Pref-1 immunofluorescent staining (100×).

Immunofluorescence staining with an anti-Pref-1 antibody showed that the cells shared about 100% positive reaction for Pref-1 (Fig 3), suggesting that the isolated cells were indeed porcine intramuscular preadipocytes.

### Subcellular localization analysis of pFTO

**Fig 4:**
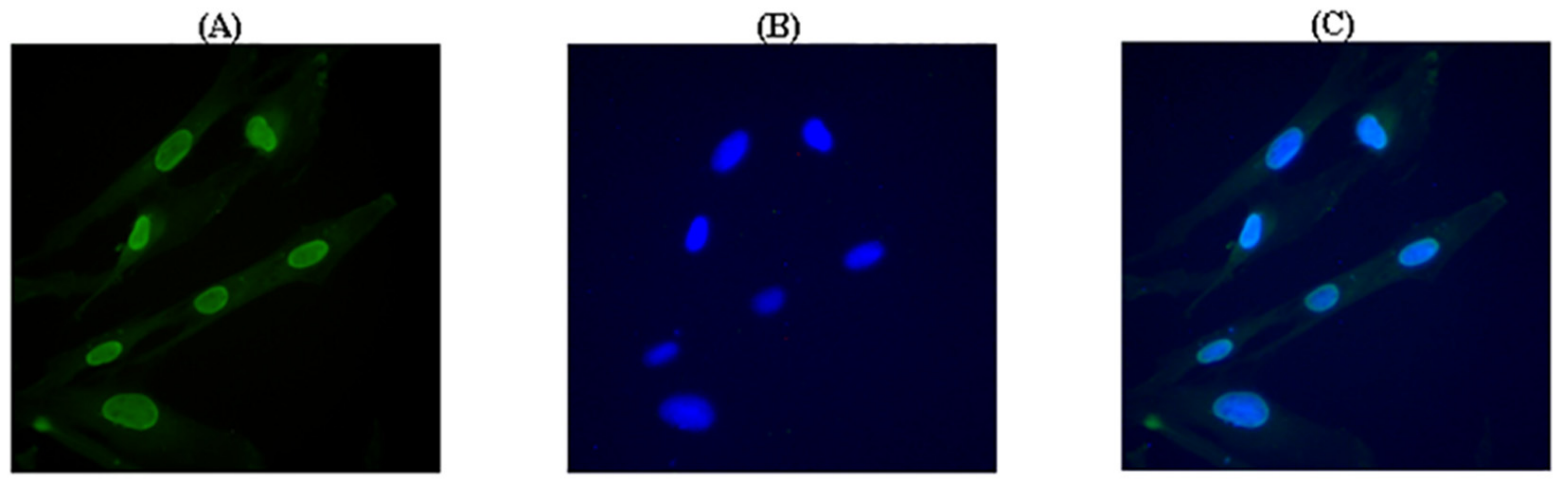
Subcellular localization of pFTO in porcine intramuscular preadipocytes using an indirect fluorescent immunocytochemical technique. (A) Endogenous pFTO protein in porcine intramuscular preadipocytes stained first with the anti-pFTO polyclonal antibody and then with FITC-conjugated goat anti-mouse IgG (green). (B) The nucleus marked with DAPI (blue). (C) The merged image.

We used the polyclonal pFTO antiserum to investigate its subcellular localization in porcine intramuscular preadipocytes by indirect fluorescent immunocytochemistry. Cell nuclei were visualized by DAPI staining. As shown in Fig 4, the pFTO protein was localized predominantly in the nucleus.

### Effect of pFTO on porcine intramuscular predipocytes proliferation

**Fig 5:**
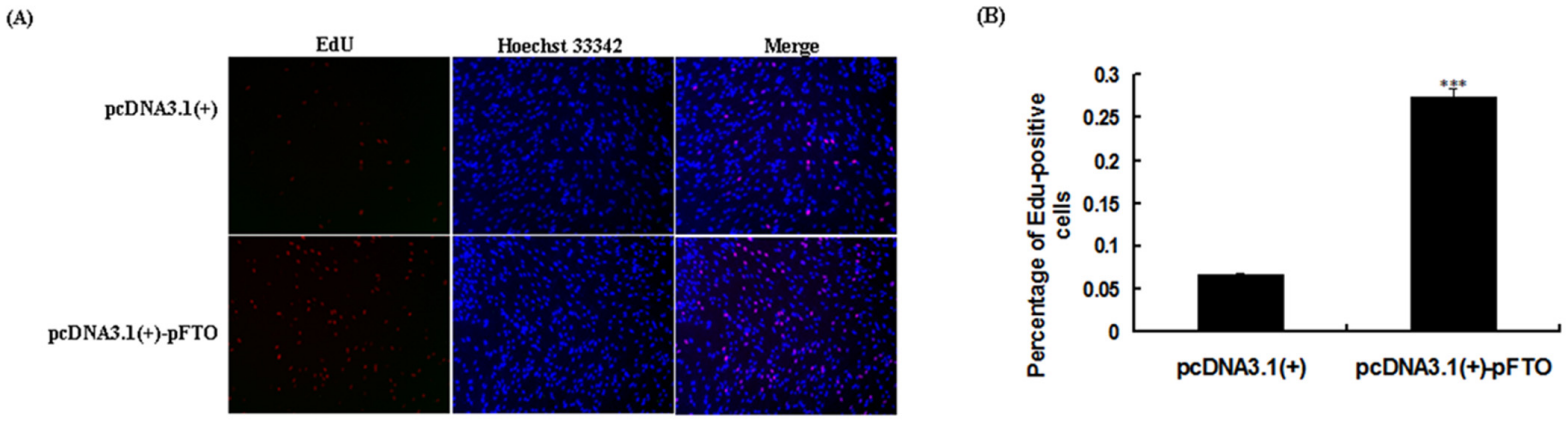
Effect of pFTO on the proliferation of porcine intramuscular preadipocytes. Porcine intramuscular preadipocytes were seeded in a 24-well plate at a density of 1 × 10^4^ cells/well. When the cells reached about 50% confluence, 0.5 μg of the plasmid pcDNA3.1(+)-pFTO or the empty vector pcDNA3.1(+) was transfected. (A) Cell proliferation was evaluated by EdU proliferation assay after 24 h of transfection. The Click-it reaction revealed EdU staining (red). The cell nuclei were stained with Hoechst 33342 (blue). (B) The percentage of EdU-positive cells was quantified. Results are presented as mean ± SE (n = 8). ***P < 0.001 as compared with the control group.

To examine the effect of pFTO on porcine intramuscular preadipocytes proliferation, the pcDNA3.1(+)-pFTO plasmid was transfected into porcine intramuscular preadipocytes. As shown in Fig 5, compared with the control group, overexpression of pFTO significantly accelerated proliferation of porcine intramuscular preadipocytes (*P* < 0.001).

### Expression profile of *pFTO* mRNA in differentiating porcine intramuscular preadipocytes

**Fig 6:**
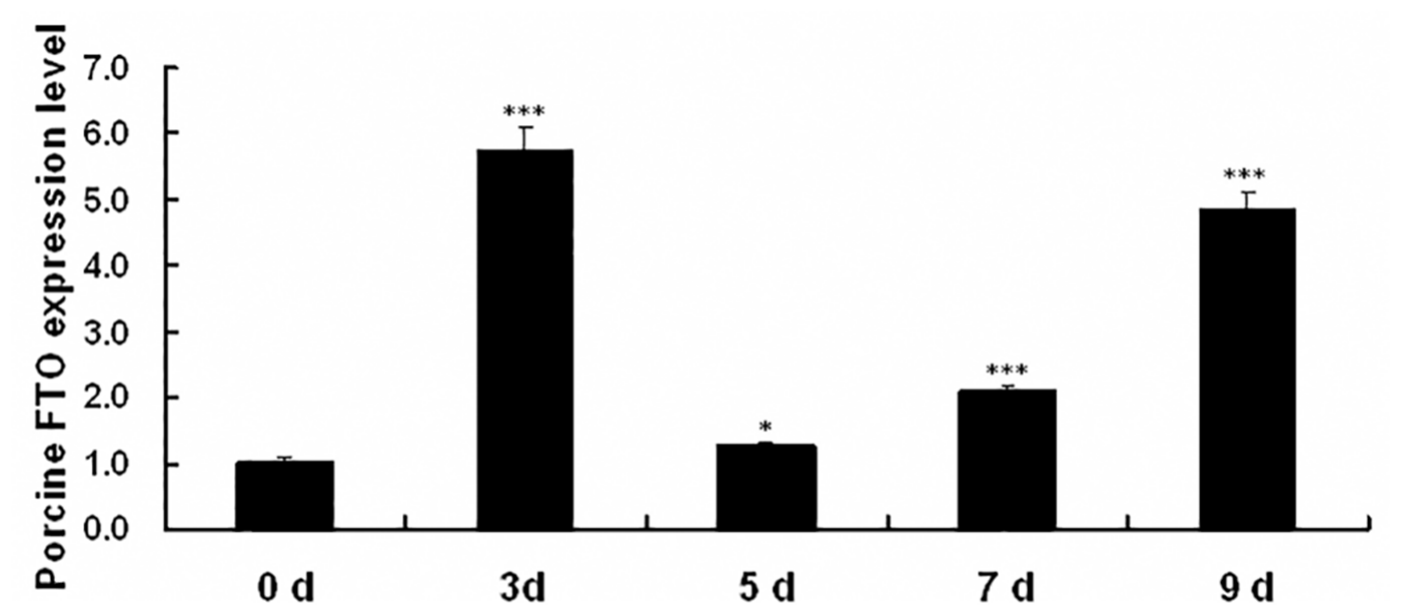
Expression pattern of *pFTO* mRNA during porcine intramuscular preadipocytes differentiation. RNA was extracted from the differentiating porcine intramuscular preadipocytes on the days 0, 3, 5, 7 and 9. *pFTO* mRNA expression was analyzed by real-time quantitative PCR. Data were the mean and SE from three independent experiments. *P < 0.05, ***P < 0.001.

To investigate the role of pFTO in fat deposit, its transcription profile during adipogenic differentiation was first analyzed using porcine intramuscular preadipocytes. Primary porcine intramuscular preadipocytes were induced to differentiate for 9 days. As shown in Fig 6, the mRNA expression of *pFTO* was initial upregulated with the highest fold upregulation at day 3. The *pFTO* mRNA expression then declined, but was observed to be upregulated subsequently at day 7 and persisted for 9 days.

### Effect of pFTO on differentiation of porcine intramuscular preadipocytes

**Fig 7:**
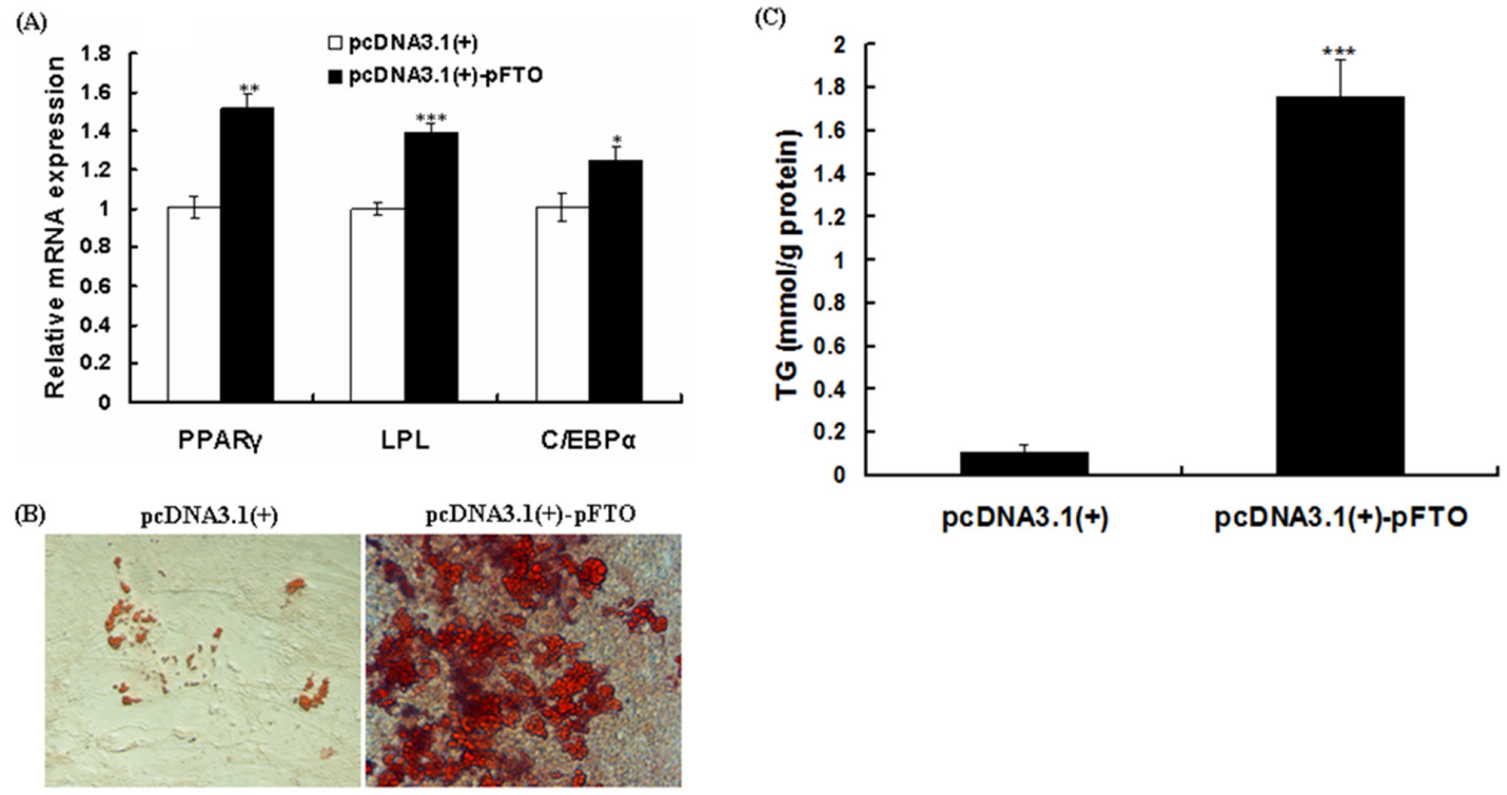
Overexpression of pFTO promotes adipogenesis and lipid accumulation. Porcine intramuscular preadipocytes were transfected with 0.5 μg of the recombinant plasmid pcDNA3.1(+)-pFTO or the empty vector pcDNA3.1(+) at the density of 90%. Cell differentiation was initiated as described in materials and methods section. (A) mRNA expression levels of *PPARγ*, *C/EBPα*, *LPL* and *FAS* were detected by real-time quantitative PCR at day 8 of differentiation. The results are represented as mean ± SE (n = 3). *P < 0.05, **P < 0.01, ***P < 0.001. (B) Intracellular lipid was stained with Oil red O at day 8 of differentiation (×400). (C) TG content was determined at day 8 of differentiation. Values are mean ± SE (n = 3). ***P < 0.001.

To test if pFTO regulates adipogenic differentiation of porcine intramuscular preadipocytes, pFTO was overexpressed by transfection of pcDNA3.1(+)-pFTO plasmid and then the cells were induced to differentiate for 8 days. Overexpression of pFTO increased the expression of the peroxisome proliferators-activated receptor γ (PPARγ), CCAAT/enhancer binding protein α (C/EBPα), fatty acid synthase (FAS) and lipoprotein lipase (LPL) (Fig 7A), suggesting that upregulation of pFTO was necessary for adipogenic differentiation. Meanwhile, Oil red O staining (Fig 7B) and TG content analyses (Fig 7C) showed that overexpression of pFTO significantly increased the lipid droplets numbers and larger droplet sizes.

## Discussion

In this study, we used the purified recombinant pFTO protein to immune Kunming mice and produced anti-FTO antiserum. The polyclonal antibody against the pFTO protein binded specifically to pFTO, which indicated that the antibody was specific and sensitive as a tool for further study the subcellular localization and physiological function of pFTO. Using indirect fluorescent immunocytochemistry, here we found that the pFTO protein was localized predominantly to the nucleus of porcine intramuscular preadipocytes, consistent with previous reports [24–26]. FTO is ubiquitously expressed in different tissue of mammals, with highest levels in brain and hypothalamus [24]. Although much attention has been paid to the role of FTO in brain/hypothalamus [24, 27], studies on its other functions are emerging [28, 29]. In this study, we examined the FTO expression at the mRNA and protein levels in porcine different tissues. We found that the mRNA and protein levels of FTO were most abundant in the lung and subcutaneous adipose of DLY pigs. Lung is an important site for priming immune protection. The high level of FTO expression found in lung suggested that FTO might play a role in protective immunity. Not surprisingly, there is a study has confirmed the relationship between FTO and immune-related infectious diseases [29]. Adipose tissue is regarded as a principle site for lipid storage and also functions as an endocrine organ with an important role in adipogenesis [26, 30–33]. In Suzhong pigs, *FTO* mRNA and protein expression in backfat was significantly higher than that in other tissue [14, 15]. Together, these studies indicated that FTO might play an important role in fat deposition and would be one of the associate genes affecting meat quality traits [11].

In this study, the expression profile of *pFTO* mRNA in differentiating porcine intramuscular preadipocytes was examined. We found that the mRNA expression of *pFTO* was significantly upregulated during porcine intramuscular preadipocytes differentiation. The dramatic upregulation of *pFTO* mRNA expression in differentiating porcine intramuscular preadipocytes was biphasic. Our result was not consistent with previous findings in 3T3-L1 preadipocytes [26, 31]. Zhao et al. found that the FTO protein expression decreased during 3T3-L1 preadipocytes differentiation [31]. Zhang et al. showed that the *FTO* mRNA expression was not significant changed during 3T3-L1 preadipocytes differentiation [26].

IMF is the last fat to be deposited within the animal. Intramuscular (IM) adipocytes proliferation and differentiation display specific biological features. The lipid metabolism and secretory function of IM adipocytes differ from that of nonmuscular adipocytes [34]. Therefore, this study was undertaken to investigate the effect of porcine FTO on the differentiation of IM adipocytes isolated from porcine skeletal muscle. In this study, we demonstrated that pFTO can enhance porcine preadipocyte proliferation. Adipogenesis is well-controlled by the sequential expression of various transcription factors [35]. PPARγ and C/EBPα have been considered as hallmarks of adipogenesis [36, 37]. Moreover, LPL, secreted by adipocytes and other tissues, is a key enzyme that plays a pivotal role in lipid metabolism by regulating lipid accumulation [38]. In the present study, pFTO overexpression significantly increased mRNA levels of *PPARγ*, *C/EBPα* and *LPL* in differentiating porcine intramuscular preadipocytes, and promoted the adipogenesis as demonstrated by Oil Red O staining and triglyceride content measurements. These results are consistent with the findings of previous *in vitro* and *in vivo* studies [26, 31–33]. *In vitro* studies using FTO depletion or FTO overexpression in 3T3-L1 preadipocytes or mouse embryonic fibroblasts have revealed that FTO was associated with the mRNA levels of adipogenesis related genes such as *PPARγ*, *C/EBPα* [26, 32, 33]. *In vivo* studies using FTO overexpression have also observed the same result [32]. Furthermore, knockdown of FTO significantly impaired adipogenic capacity as demonstrated by Oil Red O staining and triglyceride content measurements [26, 31–33]. Taken together, these results suggested that pFTO promoted porcine intramuscular preadipocytes proliferation and differentiation.

In summary, we report the production of highly specific anti-pFTO polyclonal antibody and the nuclear localization of endogenous pFTO in porcine intramuscular preadipocytes. Further, we analyzed the expression patterns of the pFTO in various porcine tissues by real-time quantitative PCR and western blot analysis. We also provide the first evidence that pFTO can enhance the porcine intramuscular preadipocytes proliferation and differentiation. Therefore, pFTO may be an important and indispensable regulator target to control the body fat deposition in pigs or to treat/prevent obesity-related disorders in humans. Additional research needs to be conducted to explore signaling pathways of pFTO that regulates adipogenic differentiation in porcine intramuscular preadipocytes.
